# Engineering Oxygen Vacancies in (FeCrCoMnZn)_3_O_4‐δ_ High Entropy Spinel Oxides Through Altering Fabrication Atmosphere for High‐Performance Rechargeable Zinc‐Air Batteries

**DOI:** 10.1002/gch2.202300199

**Published:** 2023-11-24

**Authors:** Cagla Ozgur, Tuncay Erdil, Uygar Geyikci, Can Okuyucu, Ersu Lokcu, Yunus Eren Kalay, Cigdem Toparli

**Affiliations:** ^1^ Department of Metallurgical and Materials Engineering Middle East Technical University Ankara 06800 Turkey; ^2^ Department of Metallurgical and Materials Engineering Eskisehir Osmangazi University Eskisehir 26040 Turkey

**Keywords:** high entropy oxides, oxygen evolution reaction, oxygen reduction reaction, oxygen vacancies, rechargeable zinc‐air batteries

## Abstract

High entropy oxides (HEOs) offer great potential as catalysts for oxygen electrocatalytic reactions in alkaline environments. Herein, a novel synthesis approach to prepare (FeCrCoMnZn)_3_O_4‐δ_ high entropy spinel oxide in a vacuum atmosphere, with the primary objective of introducing oxygen vacancies into the crystal structure, is presented. As compared to the air‐synthesized counterpart, the (FeCrCoMnZn)_3_O_4‐δ_ with abundant oxygen vacancies demonstrates a low (better) bifunctional (BI) index of 0.89 V in alkaline media, indicating enhanced electrocatalytic oxygen catalytic activity. Importantly, (FeCrCoMnZn)_3_O_4‐δ_ demonstrates outstanding long‐term electrochemical and structural stability. When utilized as electrocatalysts in the air cathode of Zn‐air batteries, the vacuum atmosphere synthesized (FeCrCoMnZn)_3_O_4‐δ_ catalysts outperform the samples treated in an air atmosphere, displaying superior peak power density, specific capacity, and cycling stability. These findings provide compelling evidence that manipulating the synthesis atmosphere of multi‐component oxides can serve as a novel approach to tailor their electrochemical performance.

## Graphical abstract

HEOs as air cathode in rechargeable Zn‐air battery.

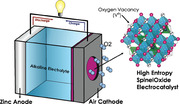

## 1 Introduction

The escalating demand for renewable energy production has generated a pressing need for grid energy storage systems that are affordable and readily accessible. Among the various energy storage devices, Zn‐air batteries have garnered significant attention as a promising next‐generation electrochemical grid energy storage solution due to their high energy densities, cost‐effectiveness, and safety.^[^ ^1^, ^2^, ^3^, ^4^, ^5^, ^6^ ^]^ In a Zn‐air battery, the cathode component extracts oxygen from the surrounding air and facilitates the electrochemical oxygen evolution reaction (OER) and oxygen reduction reaction (ORR) during the charge and discharge processes. The performance of Zn‐air batteries is heavily reliant on the kinetics of these reactions, which tend to be sluggish.^[^ ^7^, ^8^, ^9^, ^10^, ^11^, ^12^, ^13^ ^]^ Currently, state‐of‐the‐art electrocatalysts such as Pt/C, IrO_2_, and RuO_2_ are employed to mitigate the high overpotential associated with OER and ORR. However, these catalysts are scarce, expensive, and exhibit poor stability.^[^ ^14^, ^15^, ^16^ ^]^ Consequently, there is a critical need to design cost‐effective and stable bifunctional oxygen electrocatalysts that can enhance the performance of rechargeable Zn‐air batteries.^[^ ^17^ ^]^

Recently, HEOs have emerged as key players in electrochemical applications.^[^ ^18^, ^19^, ^20^, ^21^, ^22^, ^23^ ^]^HEOs are single‐phase oxides involving five or more elements in an equal molar ratio, near equal molar ratio, or non‐equimolar ratio. Specifically, HEOs involving 3d metals (Cu, Fe, Co, Ni, Zn, Mn, Cr) have demonstrated exceptional electrocatalytic activity for oxygen reactions, owing to their ability to modulate the electronic structure.^[^ ^24^, ^25^, ^26^, ^27^, ^28^, ^29^, ^30^ ^]^ For instance, Zhang et al. synthesized (CoNiMnZnFe)_3_O_3.2_ that presents a 336 mV overpotential and 47.5 mV Tafel slope.^[^ ^31^ ^]^ Spinel structured (FeCoNiCrMn)_3_O_4_ was synthesized by Duan et al. and shows low overpotential (288 mV) as an OER catalyst.^[^ ^32^ ^]^

Furthermore, the presence of oxygen vacancies exerts a substantial influence on diverse material properties, such as the electronic structure, ionic/electronic conductivity, and magnetic characteristics.^[^ ^33^, ^34^ ^]^Prior investigations have conclusively demonstrated that an optimum quantity of oxygen vacancies plays a beneficial role in enhancing the OER and ORR activity of transition metal oxides.^[^ ^35^, ^36^ ^]^ This improvement is achieved by inducing significant alterations in the bulk properties, such as energy levels and conductivity, as well as the surface properties and molecular adsorption.^[^ ^37^, ^38^ ^]^

A wide range of methodologies has been utilized to introduce oxygen vacancies in metal oxides, encompassing both in‐synthesis procedures and post‐synthesis treatments.^[^ ^39^, ^40^, ^41^ ^]^These methods include thermal treatment, reduction processing, cation/anion doping, plasma treatment, as well as other technologies such as laser, flame, exfoliation, and template strategies. Moreover, oxygen vacancies can also be generated by substituting high‐valence ions in metal oxides with low‐valence ions or by incorporating dopants that lower the formation energy of oxygen vacancies.^[^ ^42^, ^43^ ^]^

Inspired by the above discussions, herein we aim to synthesize an oxygen vacancy‐rich spinel (FeCrCoMnZn)_3_O_4‐δ_ HEO using a fast and efficient one‐step production protocol. To achieve this, we employed the co‐precipitation method, followed by sintering under air and vacuum atmospheres. By transitioning from an air to a vacuum environment during synthesis, we were able to implement a streamlined production protocol for generating oxygen vacancies. The increased concentration of oxygen vacancies subsequently enhanced the oxygen electrocatalytic activity of the (FeCrCoMnZn)_3_O_4‐δ_. (FeCrCoMnZn)_3_O_4‐δ_ calcinated in vacuum exhibits superior OER/ORR activity with low overpotentials compared to (FeCrCoMnZn)_3_O_4‐δ_ calcinated in the air atmosphere. Both air and vacuum‐fabricated samples were utilized as air cathodes on a homemade Zn‐air cell. Remarkably, a Zn‐air battery with a (FeCrCoMnZn)_3_O_4‐δ_ calcinated in vacuum shows a peak power density of 102 mW cm^−2^, specific capacity of 576.07 mA h g^−1^ at 5 mA cm^−2^ current density, and outstanding cycling stability comparing to battery produced with (FeCrCoMnZn)_3_O_4‐δ_ calcinated in air.

## 2 Results and Discussion

### 2.1 Characterization of the Crystal Structure and the Morphology

**Figure 1:**
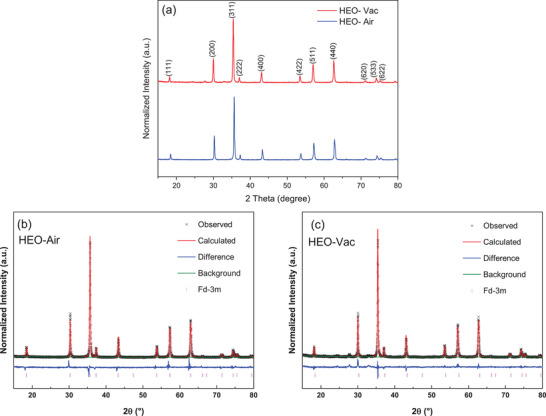
a) XRD patterns of (FeCrCoMnZn)_3_O_4‐δ_ HEO calcinated in the air (HEO‐Air) and vacuum (HEO‐Vac). b) Rietveld refinement analysis for the XRD of HEO‐Air. c) Rietveld refinement analysis for the XRD of HEO‐Vac.

The X‐ray diffraction (XRD) patterns of the HEO‐Air and HEO‐Vac are shown in **Figure** **1**a. XRD patterns revealed that both HEO‐Air and HEO‐Vac are single‐phased since there is no other peak related to the impurities, i.e., oxides or hydroxides of Fe, Cr, Co, Mn, and Zn. Rietveld Refinement was performed to understand the effect of the synthesizing environment on the change in the lattice structure (Figure 1c,d). The space groups of these samples were confirmed by Rietveld refinement analysis with considerably low χ^2^ values, as shown in Tables S1 and S2 (Supporting Information). HEO‐Air and HEO‐Vac have both spinel cubic crystal structures with a space group of $Fd\bar{3}m$ (ICDD #04‐024‐0120). The lattice parameters of HEO‐ Air and HEO‐Vac were calculated as 8.3621 and 8.3520 Å, respectively. Thus, there is a decrease in the lattice parameter in the cell volume due to the oxygen vacancy formation.

**Figure 2:**
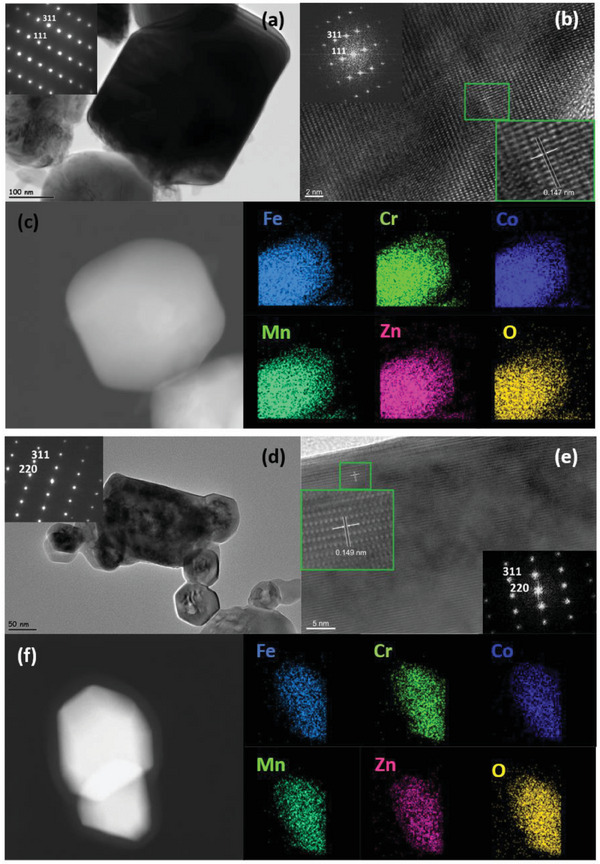
a) Bright‐field (BF) transmission electron microscopy (TEM) image of HEO‐Vac. The inset shows the selected area electron diffraction (SAED) pattern of the given crystal from <211> zone axis. b) HRTEM image of HEO‐Vac and the corresponding FFT pattern (inside) validate the Fd3m (227) space group from the <211> zone axis. c) HADDF image of HEO‐Vac and corresponding EDS element mapping of Fe, Cr, Co, Mn, Zn, and O. d) Bright‐field TEM image of HEO‐Air and the corresponding SAED pattern (inside) from the <211> zone axis. e) HRTEM image of HEO‐Air and corresponding FFT pattern (inside) from the <211> zone axis (f) HADDF image of HEO‐Air and corresponding EDS element mapping of Fe, Cr, Co, Mn, Zn, and O for HEO‐Air.

Bright‐field transmission electron microscope (TEM) image and the corresponding selected area electron diffraction (SAED) pattern from <211> zone axis for the HEO‐Vac is shown in **Figure** **2**a. The HEO particle seen in this image has an approximate particle size of 400 nm. Investigation of this crystal from <211> zone axis further validates the spinel cubic structure. High‐resolution transmission electron microscopy (HRTEM) analysis shows the lattice fringes which are also characteristic of the crystalline structure. FFT data for this crystal from <211> zone axis also matches with the $Fd\bar{3}m$ (227) space group with a d‐spacing of 0.147 nm for the (440) plane (Figure 2b). Combining these results with the Rietveld analysis made for the XRD proves that the structure of HEO‐Vac is spinel. Moreover, to investigate the distribution of the elements inside the HEO‐Vac particle, high‐angle annular dark field (HADDF) imaging and the energy dispersive spectroscopy (EDS) elemental mapping for Fe, Cr, Co, Mn, Zn, and O were done in STEM mode. The results are given in Figure 2c. EDS mapping revealed that the elements of HEO‐Vac are homogeneously distributed in the structure. Also, the same level of bright contrast inside the particle in the HAADF image indicates the homogeneous distribution of the elements. All the TEM analyses for the HEO‐Vac validate the XRD results as giving the single‐phase homogeneous solid solution of HEO‐Vac with a spinel cubic structure (227).

The same set of characterizations was also performed for the HEO‐Air powders by TEM. The bright‐field TEM image and the corresponding SAED pattern from <211> zone axis for the HEO‐Air powders are shown in Figure 2d. The particle size is around 200 nm. The corresponding SAED pattern for HEO‐Air is also in agreement with the Rietveld refinement of the XRD results, which proves the $Fd\bar{3}m$ (227) symmetry for these particles. The HRTEM image and the corresponding FFT pattern from <211> further validate the spinel cubic crystal structure with a d‐spacing of 0.149 nm for the (440) plane in Figure 2e. The results show the homogeneous distribution of the elements in the relative HAADF image, and the EDS elemental mapping are given in Figure 2f.

**Figure 3:**
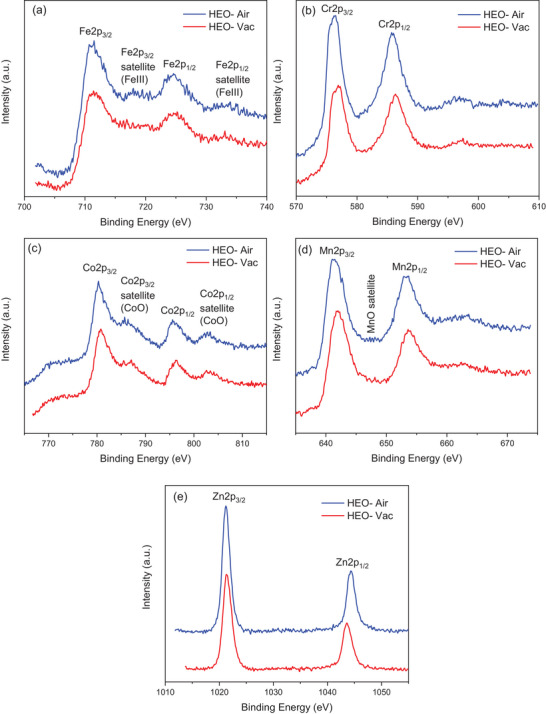
XPS spectra of (a) Fe 2p (b) Cr 2p (c) Co 2p (d) Mn 2p (e) Zn 2p for HEO‐Air and HEO‐Vac powders.

**Figure 4:**
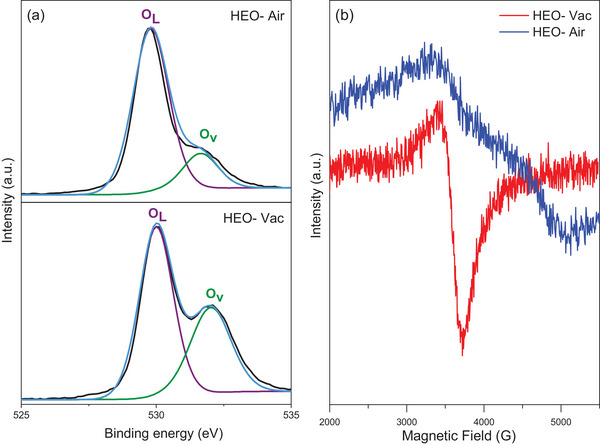
a) XPS O 1s spectra for HEO‐Air and HEO‐Vac powders, b) EPR spectrum of HEO‐Air and HEO‐Vac.

**Figure** **3** shows the X‐ray photoelectron spectroscopy (XPS) core level spectra of Fe2p, Cr2p, Co2p, Mn2p, and Zn2p (See Figure S1, Supporting Information, for the deconvolution of XPS spectra for Fe2p, Cr2p, Co2p, Mn2p, and Zn2p). Since the Co LMM peak overlaps with the deconvolution of Fe2p due to the Co LMM peak overlap at the Fe2p region, it is difficult to deconvolute Fe2p spectra. However, in Figure 3a the peaks representing the Fe 2p_3/2_, Fe 2p_3/2_ satellite, Fe 2p_1/2_, and Fe 2p_1/2_ satellite can be seen located at the binding energies of 711, 718, 724, and 732 eV respectively. The satellite from Fe^+3^ is visible in the sample calcined in air, while it disappears in the spectra of HEO‐Vac.^[^ ^17^ ^]^ Figure 3b presents Cr 2p spectra and peaks located at a binding energy of 586 and 576.0 eV representing the Cr 2p_1/2_ and Cr 2p_3/2_ signals respectively, indicating the state of Cr^+3^ presence in both air and argon synthesized atmosphere. In Figure 3c Co2p spectra (Co 2p_3/2_, Co 2p_3/2_ satellite, Co 2p_1/2_, and Co 2p_1/2_ satellite peaks located at 780, 786, 796, and 802 eV binding energies respectively) exhibit strong satellite of Co^+2^, implying both air and vacuum‐synthesized HEOs have the same state of Co. The XPS results in Figure 3d exhibit the Mn2p spectra with peaks located at binding energies of 641, 645, and 653 eV that represent Mn 2p_3/2_, MnO satellite, and Mn 2p_1/2_, respectively. Mn 2p XPS spectra suggest that MnO is present in both air and vacuum‐calcined samples, though Mn_2_O_3_ may also exist in the HEO‐Vac.^[^ ^44^ ^]^ The Zn 2p core level spectra are given in Figure 3e which implies the Zn+2 state in all samples with Zn 2p3/2 and Zn 2p3/2 peaks located at binding energies of 1021 and 1045 eV respectively.^[^ ^45^ ^]^ In **Figure** **4**a, fitted O 1s XPS spectra of HEO‐Air and HEO‐Vac are shown, which exhibit the lattice oxygen (OL), and oxygen vacancy (OV) at binding energies of 529 and 532 eV. In the case of HEO‐Vac, the OV peak accounts for 43.58% of the overall O 1s XPS spectra peak area, whereas for HEO‐Air, the OV peak constitutes 26.13% of the total area. Hence, as the oxygen amount in the synthesis environment decreases, the amount of oxygen vacancies increases in the structure which is seen in the XPS core level spectra of O1s in **Figure** **4**a.

The electron paramagnetic resonance spectroscopy (EPR) was applied to examine further and compare the oxygen vacancy content of the HEO‐Air and HEO‐Vac. The oxygen vacancy content of the electrocatalysts was determined by the EPR signal when *g* = 2.005 (Figure 4b). It is clearly seen that the EPR peak of HEO‐Vac is stronger than the peak of HEO‐Air which indicates that HEO‐Vac has more oxygen vacancy.

### 2.2 Electrochemical Performance of the Electrocatalysts

**Figure 5:**
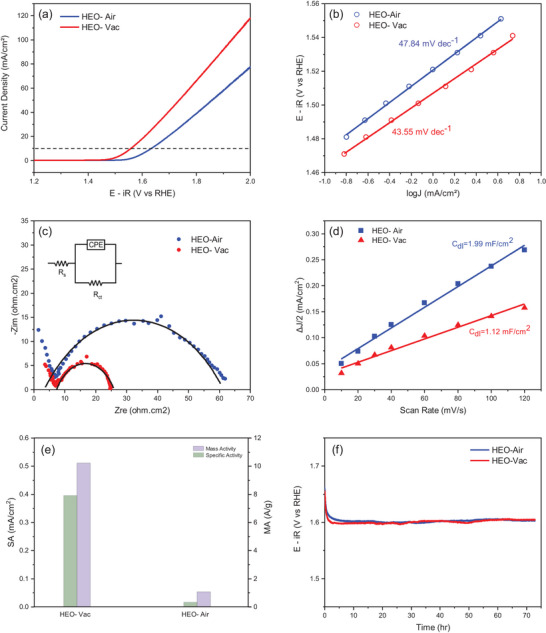
HEO‐Air and HEO‐Vac's (a) OER activity curves (b) Tafel plots that obtained from steady–state measurements (c) EIS (d) Electric double layer capacitance (e) Mass activity and the specific activity (f) Galvanostatic stability curve at a current density of 10 mA cm^−2^.

The electrocatalytic activity of the HEO‐Air and HEO‐Vac were measured with a standard three‐electrode system in a rotating disc electrode (RDE). Linear Sweep Voltammetry (LSV) was applied to the HEO‐Air and HEO‐Vac using an Ag/AgCl reference electrode, and then, the potentials were converted according to the reversible hydrogen electrode (RHE). Additionally, LSV curves were standardized with the glassy carbon electrode's geometric area, which is 0.0707 cm^2^ as shown in **Figure** **5**a. The overpotentials of HEO‐Air and HEO‐Vac are found to be 404 and 329 mV at 10 mA cm^−2^ current density, respectively. Notably, the overpotential of the HEO‐Vac is significantly lower than the HEO‐Air, which can be associated with higher oxygen vacancy content.^[^ ^46^, ^47^ ^]^ To compare the reaction kinetics of electrocatalysts Tafel plots were obtained. As seen in Figure 5b, the Tafel slopes of HEO‐Air and HEO‐Vac are 47.84 and 43.55 mV dec^−1^ respectively. Hence, HEO‐Vac has faster reaction kinetics with a lower Tafel slope as compared to HEO‐Air. Electrochemical impedance spectroscopy (EIS) which gives the charge transfer rate of the electrocatalysts was performed. Figure 5c shows the fitted EIS data with the charge‐transfer resistance (*R*_ct_), solution resistance (*R*_s_), and a constant‐phase element (CPE). The charge transfer resistance of HEO‐Air is 56.3 Ω, whereas the resistance of HEO‐Vac is 18.34 Ω. This result demonstrates that the conductivity of HEO‐Vac is higher than the HEO‐Air, revealing that HEO‐Vac has a better electrocatalytic activity for the OER due to faster charge transfer. Electrochemically active surface area (ECSA) of the HEO‐ Air and HEO‐ Vac were calculated with the double layer capacitance (*C*_dl_). *C*_dl_ of the HEO‐ Air and HEO‐ Vac was calculated as 1.99 and 1.12 mF cm^−2^, respectively (Figure 5d). Since HEO‐Vac has better electrocatalytic activity as compared to HEO‐Air, it may be envisioned that the superior electrocatalytic activity of HEO‐Vac is related to its better intrinsic activity.^[^ ^48^ ^]^ To understand the intrinsic activity, we calculated the mass activity (MA) and the specific activity (SA) of the HEO‐Air and HEO‐Vac. The mass activity was calculated from the mass loading of the electrocatalysts, whereas the specific activity was found with the Brunauer–Emmet–Teller (BET) specific area (See Tables S3 and S4, Supporting Information). Mass activity and the specific activity of the HEO‐Vac were found to be drastically higher than the HEO‐ Air as shown in Figure 5e. MA of HEO‐Vac (10.19 A g^−1^) and SA of HEO‐Vac (0.39456 mA cm^−2^) exceed the MA (8.87 A g^−1^) and SA (0.06224 mA cm^−2^) of benchmark electrocatalyst RuO_2_.^[^ ^49^ ^]^

Galvanostatic tests were applied to the HEO‐Air and HEO‐Vac in a constant current density of 10 mA cm^−2^ to investigate the long‐term stability. Figure 5f shows that both HEO‐Air and HEO‐Vac have excellent stability.

**Figure 6:**
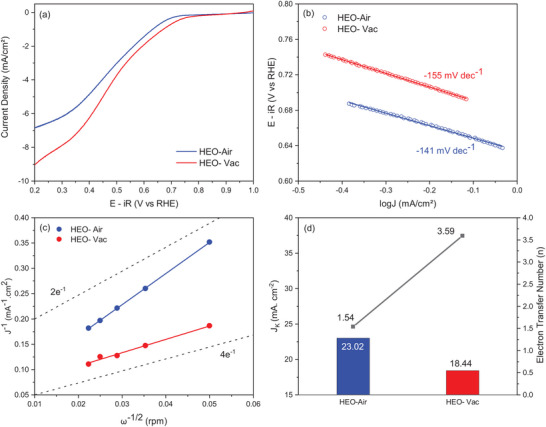
a) ORR activity curves of HEO‐Air and HEO‐Vac. b) Tafel plots of HEO‐Air and HEO‐Vac obtained from steady–state measurements. c) Koutecky–Levich plots of HEO‐Air and HEO‐Vac at 0.15 V obtained from the rotating disk electrode data in Figure S2 (Supporting Information). d) Kinetic current density (*J*_k_), and the electron transfer number (*n*) for HEO‐ Air and HEO‐Vac.

The oxygen reduction reaction activity of the HEO‐Air and HEO‐Vac are shown in **Figure** **6**. LSV curves were obtained, and at −1 mA cm^−2^ current density the potentials for HEO‐Air and HEO‐Vac are 0.63 and 0.67 V, respectively (Figure 6a). Hence, the ORR activity of HEO‐Vac is slightly higher than the HEO‐Air. From the Tafel plots, Tafel slopes are found, and it is stated as −141 mV dec^−1^ for HEO‐Air and −155 mV dec^−1^ for HEO‐Vac (Figure 6b).

The electron transfer number and the kinetic current densities of the electrocatalysts were calculated by using the Koutecky–Levich (K–L) equation. To obtain K–L plots, with RDE LSV was conducted between 1.2 and 0.14 V versus RHE in different rotation speeds (400, 800, 1200,1600, and 2000 rpm (Figure S2a,b, Supporting Information) with 5 mV s^−1^ scan rate. As the rotational speed of the working electrode increases, the reduction current increases due to the improvement in the mass transport at the working electrode surface. Diffusion of the oxygen in the electrolyte to the surface of the electrocatalyst is enhanced by the increase in rpm. HEO‐Vac consistently exhibits a higher reduction in current density in constant potential and rotational speed. K–L plots of the HEO‐Air and HEO‐Vac at different reduction potentials (0.50, 0.40, 0.30, 0.25, 0.20, and 0.15 V vs RHE) are given in Figure S2c,d (Supporting Information). The K–L plots display linearity which proves that ORR has first‐order kinetics. At low voltage (0.15 V vs RHE) the electron transfer number (n) was calculated as 3.59 and 1.54 for HEO‐Vac and HEO‐Air, respectively (Figure 6c). Since the electron transfer number of HEO‐Vac is closer to 4, it can be concluded that for HEO‐Vac 4‐electron ORR process is dominant while the HEO‐Air 2‐electron ORR process is dominant.^[^ ^50^, ^51^ ^]^ Oxygen vacancies in the structure of the HEO‐Vac enable easier charge transfer and oxygen gas adsorption, which results in better oxygen reduction performance of HEO‐Vac. The reaction kinetics of the HEO‐Air and HEO‐Vac, kinetic current densities are determined as 23.02 mA cm^−2^ for HEO‐Air and 18.44 mA cm^−2^ for HEO‐Vac (Figure 6d).

The bifunctional index (BI) was calculated from the potential gap between −1 and 10 mA cm^−2^ current densities. BI implies the catalysts’ high OER and ORR activities in an alkaline media. In this study, the BI of the HEO‐Air is 1.0 V, whereas the BI of the HEO‐Vac is 0.89 V. Hence, the BI of the HEO‐Vac is lower (better) than the HEO‐Air. As a result, HEO‐Vac shows high electrocatalytic activity in both OER and ORR.

### 2.3 Materials Characterization after the Electrochemical Stability Test

**Figure 7:**
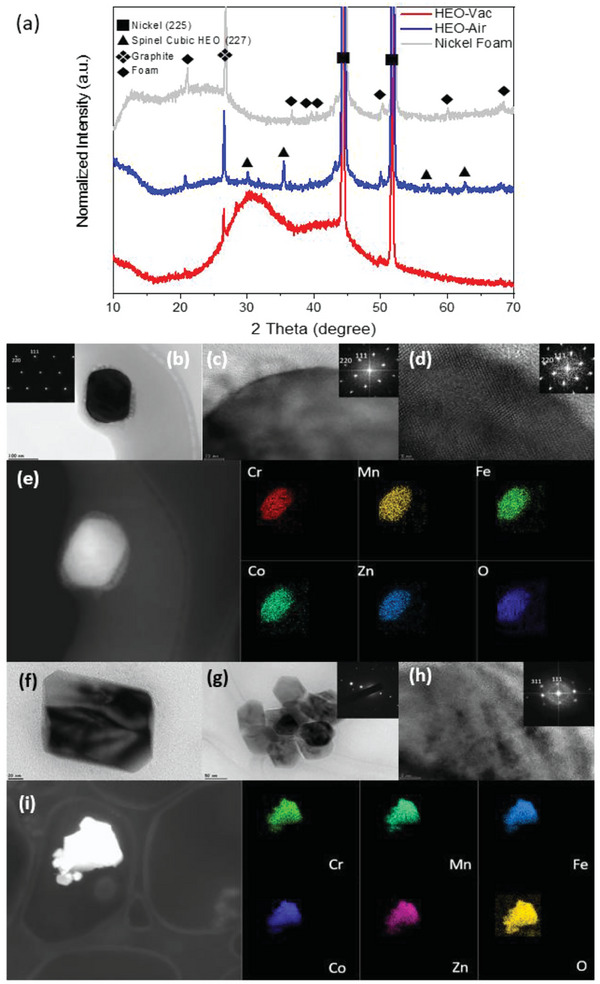
After the electrochemical stability test (a) XRD results of the HEOs and the carbon foam on nickel. b) Bright‐field (BF) transmission electron microscopy (TEM) image of the (FeCrCoMnZn)_3_O_4‐δ_ HEO‐Vac particle. The inset shows the corresponding selected area electron diffraction (SAED) pattern from <110> zone axis for the given particle. c,d) HRTEM images of the HEO‐Vac particle. The insets are the FFT signals for the given particle from the <110> zone axis. e) HAADF image and the corresponding EDS elemental mapping for the HEO‐Vac particle. f,g) Bright‐field TEM images of the HEO‐Air particles. The inset shows the corresponding SAED pattern from the agglomerated HEO‐Air particles. h) HRTEM analysis of the HEO‐Air particle. The inset shows the corresponding FFT signal for the given particle from <211> zone axis. i) HAADF image and EDS elemental mapping for the given HEO‐Air particle.

After the stability experiments, XRD and TEM characterizations were carried out to examine the potential structural and chemical changes of the HEO particles. The XRD analyses in **Figure** **7**a summarize the structural changes. The necessity of carbon foam‐coated nickel usage during the stability experiments is the reason for the intense nickel, graphite, and foam peaks in the XRD data. After the elimination of these peaks, all the remaining peaks were in good agreement with the $Fd\bar{3}m$ (227) spinel cubic structure. Therefore, no phase transformation occurred for the HEO‐Air and HEO‐Vac particles during the stability tests.

The bright‐field TEM image of the HEO‐Vac powder in Figure 7b has an average particle size of 200 nm. The size of these particles is almost the same as the size of the particles before the stability experiments. The corresponding selected area electron diffraction pattern from <110> zone axis matches the spinel cubic crystal structure. The HRTEM images for the after‐stability HEO‐Vac particles show no amorphization or structural changes after the electrochemical tests. The given particle in Figure 7c,d is still completely crystalline, and the corresponding FFT signals validate the spinel cubic structure with $Fd\bar{3}m$ (227) space group. Several analyses under STEM mode give insight into the possible chemical changes in Figure 7e, HAADF image of the after‐stability HEO‐Vac specimen shows a single bright contrast inside. Therefore, one expects no chemical changes compared to the initial as‐synthesized HEO‐Vac particles. EDS mapping of the given particle validates this theory and shows a homogeneous distribution of the elements Cr, Mn, Fe, Co, Zn, and O. After all these analyses, we can conclude that the single‐phase solid solution spinel cubic structure with the homogeneous distribution of the components has remained still.

Investigation of any potential change for the HEO‐Air particles after the electrochemical tests were done with the same route. First, the bright‐field TEM images of HEO‐Air particles in Figure 7f,g show the same morphology as the initial ones. Furthermore, the particles still have a single‐phase crystal structure. The HRTEM analysis from these HEO‐Air particles shows an unchanged crystalline nature after the stability tests in Figure 7h. The corresponding FFT signal from <211> zone axis validates that the crystalline structure of the analyzed particle has remained the same as $Fd\bar{3}m$ (227) spinel cubic under electrochemical activity. After the structural investigation of after‐stability HEO‐Air particles, the chemical distribution of the components was observed by HAADF and the EDS elemental mapping in Figure 7i. There is no segregation of the elements after the experiments. The structure and the homogeneity of the HEO‐Air particles were also preserved during the stability tests.

### 2.4 Evaluation of the Zinc‐Air Battery Performance

The Zn‐air battery consists of a Zn plate anode, high entropy spinel oxide cathode, and 6 m KOH electrolyte with 0.2 m Zn(OAc)_2_ additive. The open‐circuit potential (OCP) of the HEO‐Vac is 1.642 V, which is slightly higher than the OCP of HEO‐Air (1.619 V).

**Figure 8:**
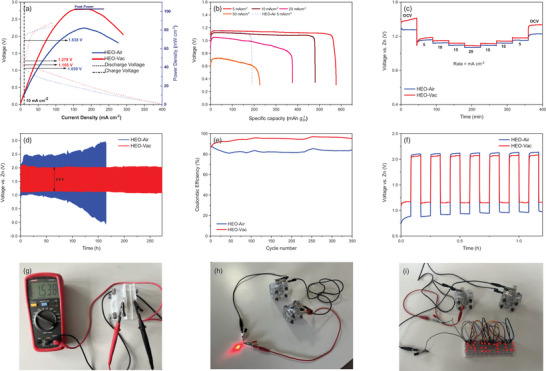
Zn‐Air batteries with HEO‐Vac, and HEO‐Air electrocatalysts as air cathodes’ a) Charge and discharge polarization curves, and corresponding peak power density plots. b) Specific capacities at 5, 10, 20, and 50 mA cm^−2^ current densities. c) Rate capability study from 0 to 20 mA cm^−2^ and then back to 0 mA cm^−2^. d) Cyclic charge–discharge curves at 5 mA cm^−2^. e) Coulombic efficiencies at 5 mA cm^−2^. f) Durability performance in the first seven cycles of cyclic charge‐discharge. g) Open circuit voltage of HEO‐Vac Zn‐Air battery. h,i) Digital photographs of working HEO‐Vac Zn‐Air battery.

**Figure** **8**a shows the charge and discharge polarization curves of the zinc‐air battery with HEO‐Vac and HEO‐Air cathodes. The Zn‐air battery utilizing the HEO‐Vac cathode exhibits a lower charging voltage and higher discharge voltage in comparison to the Zn‐air battery with the HEO‐Air cathode. This observation suggests that the HEO‐Vac catalyst displays a reduced voltage gap between the oxygen evolution reaction and oxygen reduction reactions during charge and discharge processes, thereby enhancing the rechargeability of the HEO‐Vac‐based Zn‐air battery. To reach 10 mA cm^−2^ current density during the charge and discharge processes HEO‐Vac cathode requires 1.279 and 1.165 V, respectively. On the other hand, the HEO‐Air cathode requires 1.838 and 1.059 V during the charge and discharge processes, respectively (Figure 8a). The HEO‐Vac cathode demonstrates an impressive peak power density of 102 mW cm^−2^ when operating at a current density of 180 mA cm^−2^. This value surpasses the power density achieved by the HEO‐Air cathode (82 mW cm^−2^) and is comparable to the power densities reported for catalysts in existing literature.

The specific capacities at different current densities from 5 to 50 mA cm^−2^ for HEO‐Vac and the specific capacity of HEO‐Air at 5 mA cm^−2^ are shown in Figure 8b. The HEO‐Vac‐based Zn‐air battery exhibits a maximum specific capacity of 576.07 mA h g_Zn_^−1^, which corresponds to approximately 70% of the theoretical Zn capacity (820 mA h g_Zn_^−1^) when operated at a current density of 5 mA cm^−2^.^[^ ^52^, ^53^ ^]^Furthermore, it boasts a specific energy of 662.46 W h kg^−1^. Notably, the Zn‐air battery based on the HEO‐Vac catalyst demonstrates excellent high‐rate capability, delivering specific capacities of 479.89, 376.76, and 225.31 mA h g_Zn_^−1^ at current densities of 10, 20, and 50 mA cm^−2^, respectively. In contrast, the Zn‐air battery utilizing the HEO‐Air cathode only achieves a specific capacity of 190.77 mA h g_Zn_^−1^ at a discharge rate of 5 mA cm^−2^. Furthermore, to satisfy the technologically significant benchmark of 100 W h kg^−‐1^, the minimum required depth of discharge of zinc (DOD_Zn_) within a Zn–air battery should be 0.20.^[^ ^54^ ^]^ In this work, DOD_Zn_ at current densities of 5, 10, 20, and 50 mA cm^−2^ for HEO‐Vac‐based Zn‐air batteries were calculated as 0.70, 0.59, 0.46, and 0.27, respectively. Whereas DOD_Zn_ for HEO‐Air based Zn‐air battery is equal to 0.23. The rate capabilities and cycling stabilities of HEO‐Vac‐based and HEO‐Air‐based Zn‐air batteries are compared in Figure 8c. The discharge rates change from 0 to 20 mA cm^−2^ and then back to 0 mA cm^−2^ stepwise to measure the output voltage. As shown in Figure 8c, the HEO‐Vac‐based Zn‐Air battery offers a greater rate capability and higher stability. After 400 min of operation, the output voltage is 1.336 V which is 98.45% of the initial output voltage of 1.357 V. On the other hand, the Zn‐air battery based on the HEO‐Air cathode has an output voltage of 1.229 V (94.54% of the initial output voltage of 1.3 V) in the same operation time.

To understand the electrochemical durability of Zn‐air batteries, the cyclic charge‐discharge performance of the batteries was measured at 5 mA cm^−2^. Zn‐air battery with HEO‐Vac cathode shows a 2.0 V charge and 1.1 V discharge voltage during cycling with a 0.9 V voltage gap as can be seen in Figure 8d. The charge and discharge voltages of the Zn‐air battery with HEO‐Vac remain stable during 300 h of cycling, Hence, the HEO‐Vac Zn‐air battery exhibits outstanding rechargeability and durability. It shows that HEO‐Vac effectively catalyzes OER and ORR during charge and the discharge of the battery even after 300 h. In contrast, the Zn‐air battery with HEO‐Air cathode displays a 2.5 V discharge voltage, and 1.0 V charge voltage with a greater voltage gap than the HEO‐Vac Zn‐air battery in the first cycles. Then, it lost its stability after nearly 75 h as expected, since; HEO‐Air exhibits lower OER/ORR bifunctional electrocatalytic activity as discussed in Section 2.2. Hence, the Zn‐air battery with the HEO‐Vac electrocatalyst exhibits better performance than the battery with the HEO‐Air electrocatalyst. This is mainly due to the better bifunctional electrocatalytic performance of the HEO‐Vac which is attributed to the higher oxygen vacancy content in its structure.

To further understand the cycling performance of HEO‐Vac‐based and HEO‐Air‐based Zn‐air batteries Coulombic efficiency of Zn‐air batteries was measured by charging the battery at a constant current density of 5 mA cm^−2^ for 5 min and discharging the battery at a constant load of 165 Ω (at an average discharge current density of 5 mA cm^−2^) for 5 min over 350 cycles. As shown in Figure 8e, the Coulombic efficiency for the HEO‐Vac‐based Zn‐air battery remained above 87% reaching 96% on the upper end while the HEO‐Air‐based Zn‐air battery's Coulombic efficiency remained below 87% dropping to 80% on the lower end over the course of 350 cycles. Moreover, durability performance in the first seven cycles of cyclic charge‐discharge for HEO‐Air and HEO‐Vac‐based batteries is shown in Figure 8f. Charge–discharge voltages and the voltage gap difference between HEO‐Air and HEO‐Vac‐based Zn‐air batteries can be clearly seen in Figure 8f.

Beginning‐of‐life (BOL) EIS and end‐of‐life (EOL) EIS were employed to further analyze the resistive factors associated with the operation of the HEO‐Air and HEO‐Vac‐based zinc‐air battery, as illustrated in Figure S3 (Supporting Information) via the Nyquist plots, in combining with an equivalent circuit. Nyquist plots are modeled using an equivalent circuit consisting of five elements: *R*_s_, *Q*_int_, *R*_int_, *Q*_dl_, and *R*_ct_, consistent with prior research on Zn–air batteries. *R*_s_ and *R*_int_ denote the resistances attributed to the electrolyte and contact, as well as the resistance at the solid–liquid electrolyte interface, respectively. *Q*_int_ and *Q*_dl_ represent the capacitance characteristics at the electrode‐electrolyte interface within the battery. Lastly, *R*_ct_ signifies the charge‐transfer resistance experienced at the air cathode during electrochemical reactions, which correlates directly with the catalyst's catalytic activity. The values of these elements were determined based on the equivalent circuit and are presented in Table S5 (Supporting Information). While the initial *R*_ct_ value in the EIS analysis for Zn‐Air battery with HEO‐Vac based electrocatalyst is higher than that of Zn‐Air battery using HEO‐Air at the beginning of their life (BOL), the end‐of‐life (EOL) results show a significant difference. In the case of HEO‐Vac, the *R*_ct_ value increases by approximately two‐fold, whereas for HEO‐Air, it experiences a more substantial increase, exceeding ten times. This indicates that the stability of the HEO‐Air electrocatalyst diminishes significantly after undergoing 100 charge‐discharge cycles at a current density of 10 mA cm^−^^2^.

In addition, the open circuit potential of the Zn‐Air battery with HEO‐Vac electrocatalyst is 1.538 V as shown in Figure 8g. As displayed in Figure 8h,i, the Zn‐Air battery with HEO‐Vac electrocatalyst in an air cathode could power a panel that is written “METU” with red LEDs. To showcase real‐world usability, we connected two Zinc‐air batteries with HEO‐Vac air cathodes in series. These batteries achieved an open circuit voltage of 2.952 V and were able to power 35 red LEDs (2.7 V each) continuously and reliably for over 48 h.

## 3 Conclusion

We successfully synthesized single‐phase, spinel crystal structured (FeCrCoMnZn)_3_O_4‐δ_ high entropy oxide under both air and vacuum fabrication atmospheres. By switching the calcination environment from air to vacuum, we observed an increased oxygen vacancy content in the (FeCrCoMnZn)_3_O_4‐δ_. HEO‐Vac outperforms HEO‐Air in terms of electrocatalytic OER and ORR activity, owing to its higher oxygen vacancy content that enhances charge transfer rates and catalytic activity. Notably, HEO‐Vac displays remarkable long‐term stability and exceptional overpotential of 329 mV at a current density of 10 mA cm^−2^. Furthermore, the BI of HEO‐Vac is 0.89 V, which can be favorably comparable with state‐of‐the‐art electrocatalysts. The battery assembled with HEO‐Vac electrocatalyst demonstrates a high peak power density of 102 mW cm^−2^, a specific capacity of 576.07 mA h g^−1^, and a specific energy of 662.46 W h kg^−1^ at 5 mA cm^−2^ current density. As compared to the Zn‐air battery with HEO‐Air electrocatalyst, the HEO‐Vac‐based Zn‐air battery displays better performance with higher capacity, durability, and efficiency. We present a novel approach to modify the structure of high entropy oxides by manipulating the synthesis environment. This method holds significant potential in enhancing the utilization of high entropy oxides in electrochemical energy storage devices.

## 4 Experimental Section

### Synthesize of High Entropy Oxide (FeCrCoMnZn)_3_O_4‐δ_

**Figure 9:**
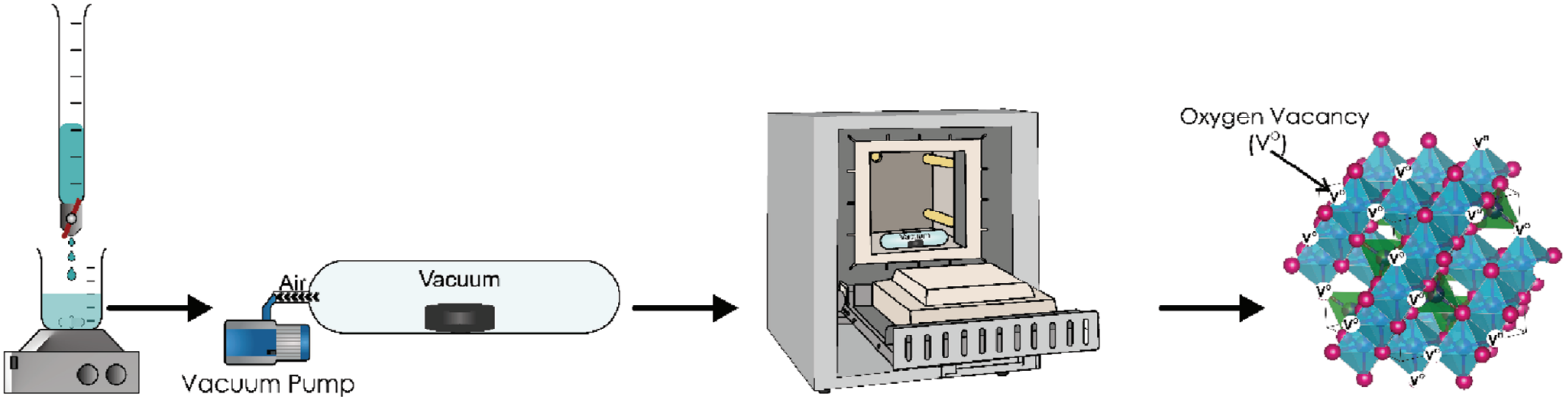
Schematic of the production steps of (FeCrCoMnZn)_3_O_4‐δ_ high entropy oxide synthesized in a vacuum.

(FeCrCoMnZn)_3_O_4‐δ_ high entropy oxide was synthesized through the co‐precipitation method. FeSO_4_·7H_2_O (Sigma–Aldrich, 99.5%), MnSO_4_·H_2_O (Sigma–Aldrich, 99%), Cr(NO_3_)_3_·9H_2_O (Sigma–Aldrich, 99%), CoSO_4_·7H_2_O (Sigma–Aldrich, 99%), and Zn(NO_3_)_2_·6H_2_O (Sigma–Aldrich, 98%) metal nitrate salts were mixed in stoichiometric amounts and dissolved in deionized water (18.2 MΩ cm). As a precipitating agent, 1 m aqueous potassium hydroxide solution was added slowly to the solution. The drying procedure was applied to the powders in an oven at 110 °C for 8 h followed by a heat treatment in air and vacuum atmospheres separately at 900 °C for 60 min. In this work, the (FeCrCoMnZn)_3_O_4‐δ_ high entropy oxides that are synthesized in air and vacuum are called HEO‐Air and HEO‐Vac, respectively. **Figure** **9** displays the experimental schematic applied to fabricate HEO‐Vac in this work.

### Materials Characterization

The crystal structure of the samples was characterized by powder X‐ray diffraction (XRD) experiments with Ni‐filtered Cu Kα radiation (*λ* = 1.5406 Å) in a 2θ range of 10°−80°. Rigaku DMAX2200 diffractometer operated at 40 kV/30 mA with ultima + theta‐theta high‐resolution goniometer is used for these experiments at Bragg Brentano geometry. The XRD patterns of HEO‐Air and HEO‐Vac were refined with the Rietveld Refinement Method using GSAS software and the EXPGUI interface. Field emission high‐resolution transmission electron microscope (HRTEM, JEOL JEM 2100F 200 kV) was used to study the microstructure and morphology of the samples. Bright field, high angle annular dark field, high‐resolution transmission electron microscopy images, EDS elemental mappings, and selected area electron diffraction (SAED) patterns of HEO‐Air and HEO‐Vac were obtained to analyze the microstructure. Specific areas of the HEO‐Air and HEO‐Vac were calculated using the Brunauer–Emmet–Teller (BET) method within the relative pressure change *P*/*P*_0_ = 0.055–0.30. Nitrogen (N_2_) adsorption–desorption isotherms obtained by Quantachrome Corporation. Autosorb‐6 was used to determine the specific areas while the degassing temperature of the samples was 300 °C for 2 h under vacuum. X‐ray photoelectron spectroscopy (XPS, PHI 5000 Versa Probe spectrometer) with Al Kα radiation was used to examine the chemical composition and the nature of the HEO‐Air and HEO‐Vac. Standard C 1s spectrum at 284.6 eV was used for the calibration of the peaks. The PHI 5000 charge compensation system was utilized to prevent localized charge accumulation. Electron paramagnetic resonance spectroscopy (EPR, Bruker ELEXSYS E580) was conducted to investigate the oxygen vacancy content of HEOs.

### Electrochemical Characterization

The electrochemical activity of HEO‐Air and HEO‐Vac electrocatalysts were analyzed with Rotating Disc Electrode (RDE, BASI) by using a GAMRY Reference 3000 potentiostat/galvanostat/ ZRA. The standard three‐electrode system with platinum wire as a counter electrode, Ag/AgCl as a reference electrode, and glassy carbon electrode (GCE) as a working electrode was used. The catalysts inks were prepared using 5 mg Super‐P carbon, 8 mg of HEO, 50 µL Nafion solution (Sigma–Aldrich, 5 wt.%), and 2 mL ethanol. Ten microliters of catalyst ink was dropped on a GCE and dried in air. The dried electrodes were tested in 0.1 m KOH solution which was saturated with O_2_ prior to and during the electrochemical tests. Linear sweep voltammetry (LSV) test was applied between 0.2 and 1.1 V versus Ag/AgCl with a 10 mV s^−1^ scan rate. All the potentials that are recorded versus Ag/AgCl were normalized to the reversible hydrogen electrode (RHE) according to the Nernst equation. To conduct Tafel analysis under steady–state conditions, the chronoamperometry (CA) technique within a potential window spanning from 0.40 to 0.65 V relative to Ag/AgCl, with increments of 0.01 V (Figure S4, Supporting Information) was utilized. Electrochemical impedance spectroscopy (EIS) was performed with AC voltage with an amplitude of 10 mV in a 10^5^–10^−2^ Hz frequency range. EIS was recorded at 1.641 V versus the RHE. Electrochemically active surface area (ECSA) was measured from the double layer capacitance (*C*_dl_), which can be calculated from the CV measurements performed at different scan rates in the non‐faradaic region.^[^ ^16^, ^55^, ^56^ ^]^ CVs were measured between 0.2 and 0.3 V versus Ag/AgCl with a scan rate of 10, 20, 30, 40, 60, 80, 100, and 120 mV s^−1^ (Figure S5, Supporting Information). The graph of scan rate versus half of the difference between anodic and cathodic current densities (Δ*J*/2 = (*J*_a_−*J*_c_)/2) was plotted at 0.25 V versus Ag/AgCl.^[^ ^57^, ^58^ ^]^ After the linear fitting, the *C*_dl_ of the HEO‐Air and HEO‐Vac were calculated from one‐half of the slope. ECSA was calculated from *C*_dl_ divided by the specific capacitance (*C*_s_) while the *C*_s_ in 1 m KOH is equal to 0.040 mF cm^−2^.^[^ ^59^ ^]^ The mass activity (A g_HEO_^−1^) and the specific activity (mA cm_HEO_^−2^) of the samples were calculated from the mass loading and the specific BET surface areas of the electrocatalysts. A galvanostatic test was applied to the HEO‐Air and HEO‐Vac to measure the long‐term stability. Throughout the test, current density was kept constant at 10 mA cm^−2^ and the potential was recorded for approximately 75 h.

To understand the ORR activity of HEO‐Air and HEO‐Vac, CV and LSV tests were applied in the same manner, except the potentials were applied between 0.1 and (−1.2) V versus Ag/AgCl. Then, Tafel plots were obtained, and the Tafel slopes of the electrocatalysts were calculated. A series of LSVs were performed at a scan rate of 5 mV s^−1^ in between 0.2 and −0.8 V versus Ag/AgCl with various rotation speeds (400, 800, 1200, 1600, and 2000 rpm) (Figure S2, Supporting Information) to evaluate the kinetic current density and the electron transfer number of HEO‐Air and HEO‐Vac using the Koutecky–Levich (K–L) equation was utilized.^[^ ^35^, ^60^, ^61^ ^]^

### Evaluation of the Zn‐Air Battery Performance

Zinc‐air batteries were assembled by using a zinc foil as an anode, HEO‐Vac and HEO‐Air electrocatalysts as a cathode, and 6.0 m KOH mixed with 0.2 m Zn(OAC)_2_ as an electrolyte. The air cathodes consist of a gas diffusion layer, HEO‐Vac and HEO‐Air electrocatalyst, and a PTFE film. Battery performances were measured with GAMRY Interface 1000 potentiostat/galvanostat/ZRA. Charge and discharge polarization curves of the HEO‐Vac and HEO‐Air‐based Zn‐Air batteries and their power densities were calculated from 0 to 200 mA cm^−2^ current densities. Batteries were fully discharged at 5 mA cm^−2^ current density to find the specific capacities and specific energies respectively by using Equations (1 and 2). In addition, for batteries assembled with HEO‐Vac electrocatalysts, specific capacities, specific energies, and depth of discharge of the Zn foil (DOD_Zn_) were calculated in 5, 10, 20, and 50 mA cm^−2^ discharge current densities, respectively (See Figure S6, Supporting Information, for voltage versus time at different current densities). In Equations (1 and 2), the symbols represent the following variables: *I* represents the applied current to the battery, *t* corresponds to the total discharge duration, *m* signifies the mass of the consumed zinc, measured in grams, and *V* denotes the voltage observed during the discharge plateau. Moreover, in Equation (3), *C* is the capacity, *A* is the area, *V* is the volume, and *ρ* is the density of the zinc foil.

(1) $$\mathrm{Specific}\;\mathrm{Capacity}\;\left(C_{\mathrm{Zn}-\mathrm{Air}}\right)=\frac{I\times t}{\;m}$$

(2) $$\mathrm{Specific}\;\mathrm{Energy}=\frac{I\times V\times t}{\;m}$$

(3) $$\begin{array}{c} {\mathrm{DOD}}_{\mathrm{Zn}}=\frac{C_{\mathrm{Zn}-\mathrm{Air}}}{C_{\mathrm{Zn}}}=\frac{A\times j\times t}{V_{\mathrm{Zn}}\times \rho_{\mathrm{Zn}}\times 819.73}=\frac{A_{\mathrm{cathode}}\times j\times t}{A_{\mathrm{Zn}}\times h_{\mathrm{Zn}}\times \rho_{\mathrm{Zn}}\times 819.73} \end{array}$$

Cycling stability and the rate capability were measured by comparing the output voltage of the Zn‐Air batteries at a rate of 0–20 mA cm^−2^ and then back to the 0 mA cm^−2^. Then, at 5 mA cm^−2^ current density cycling charge‐discharge performance of the Zn‐Air batteries was obtained. Discharge and charge time were 5 min per cycle, respectively. To calculate the Coulombic efficiencies of the batteries, cycling charge–discharge tests were conducted, during the tests the batteries were charged at 5 mA cm^−2^ for 5 min and then discharged at 165 Ω for 5 min. EIS in HEO‐Vac and HEO‐Air‐based Zn‐Air batteries were performed by maintaining a steady cell potential of 0.8 V while applying an alternating current (AC) amplitude of 20 mV. The frequency of the AC signal varies from 100 kHz down to 0.1 Hz. Beginning of life (BOL) EIS was performed before cyclic charge–discharge. Then, end‐of‐life (EOL) EIS was conducted after 100 cycles of cyclic charge‐discharge at 10 mA cm^−2^ while charge and discharge time were 10 min.

## Conflict of Interest

The authors declare no conflict of interest.

## Supporting information

Supporting Information

## Data Availability

The data that support the findings of this study are available from the corresponding author upon reasonable request.
