# Supplementary material for: Engineering Oxygen Vacancies in (FeCrCoMnZn)3O4‐δ High Entropy Spinel Oxides Through Altering Fabrication Atmosphere for High‐Performance Rechargeable Zinc‐Air Batteries
Source: Glob Chall. 2023 Nov 24;8(1):2300199. doi: 10.1002/gch2.202300199 (PMC10784197; doi:10.1002/gch2.202300199)
Supplement: Supplementary file 1 — Supporting Information [file GCH2-8-2300199-s001.pdf]

Global Challenges
Open Access

Supporting Information

for *Global Challenges.*, DOI 10.1002/gch2.202300199

Engineering Oxygen Vacancies in $(FeCrCoMnZn)_3O_{4-\delta}$ High Entropy Spinel Oxides Through Altering Fabrication Atmosphere for High-Performance Rechargeable Zinc-Air Batteries

*Cagla Ozgur, Tuncay Erdil, Uygar Geyikci, Can Okuyucu, Ersu Lokcu, Yunus Eren Kalay and Cigdem Toparli**

Engineering Oxygen Vacancies in $(FeCrCoMnZn)_3O_{4-\delta}$ High Entropy Spinel Oxides Through Altering Fabrication Atmosphere for High-Performance Rechargeable Zinc-Air Batteries

Cagla Ozgur, Tuncay Erdil, Uygar Geyikci, Can Okuyucu, Ersu Lokcu, Yunus Eren Kalay, and Cigdem Toparli *

Table S1. Rietvelt refinement data for $(FeCrCoMnZn)_3O_{4-\delta}$ synthesized in air (HEO-Air).

Sample ID				HEO-Air		
Phase				Spinel		
χ 2				1.358		
Space Group				Fd-3m		
Cell Volume (Å^3)				584.73		
Crystal Density (g cm^{-3})				5.3668		
Lattice parameter				8.3621		
**Label**	**Wyckoff site**	**x**	**y**	**z**	**Atom**	**Occupation**
Anion	32e	0.2551	0.2551	0.2551	O	1
Oct.	16d	0.5	0.5	0.5	Fe	0.2
					Co	0.2
					Cr	0.2
					Mn	0.2
					Zn	0.2
Tetr.	8a	0.125	0.125	0.125	Fe	0.2
					Co	0.2
					Cr	0.2
					Mn	0.2
					Zn	0.2

Table S2. Rietvelt refinement data for $(FeCrCoMnZn)_3O_{4-\delta}$ synthesized in vacuum (HEO-Vac).

Sample ID				HEO-Vac		
Phase				Spinel		
χ 2				1.913		
Space Group				Fd-3m		
Cell Volume ($Å^3$)				582.61		
Crystal Density (g cm^{-3})				5.3864		
Lattice parameter				8.3520		
**Label**	**Wyckoff site**	**x**	**y**	**z**	**Atom**	**Occupation**
Anion	32e	0.2555	0.2555	0.2555	O	1
Oct.	16d	0.5	0.5	0.5	Fe	0.2
					Co	0.2
					Cr	0.2
					Mn	0.2
					Zn	0.2
Tetr.	8a	0.125	0.125	0.125	Fe	0.2
					Co	0.2
					Cr	0.2
					Mn	0.2
					Zn	0.2

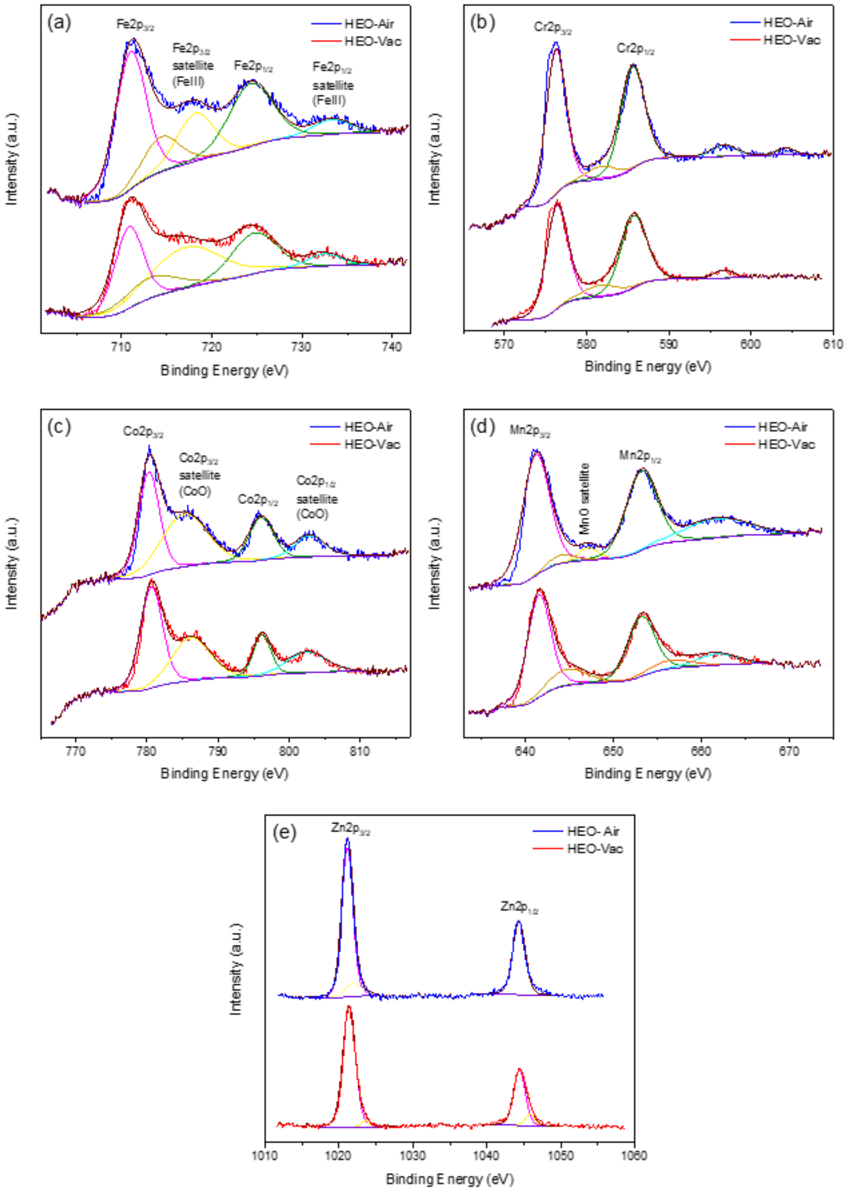

Figure S1. Deconvolution of XPS spectra for (a) Fe2p (b) Cr2p (c) Co2p (d) Mn2p, and (e) Zn2p

Table S3. BET analysis for HEO- Air

Area		7.009 m^2/g
Slope		485.9
Y-Intercept		10.95
Correlation Coefficient		0.998366
C		45.36
P/P_0	Volume [cc/g]	$1/(W((P_0/P)-1))$
0.055183	1.3273	35.21
0.087283	1.4311	53.47
0.11243	1.5133	66.97
0.16212	1.6408	94.35
0.21037	1.918	111.1
0.26148	2.091	135.5
0.31204	2.2184	163.6

Table S4. BET analysis for HEO- Vac

Area		2.583 m^2/g
Slope		1256
Y-Intercept		92.47
Correlation Coefficient		0.992367
C		14.58
P/P_0	Volume [cc/g]	$1/(W((P_0/P)-1))$
0.061216	0.3399	153.5
0.087886	0.3746	205.8
0.11292	0.4002	254.5
0.21155	0.6115	351.1

0.2624	0.6751	421.6

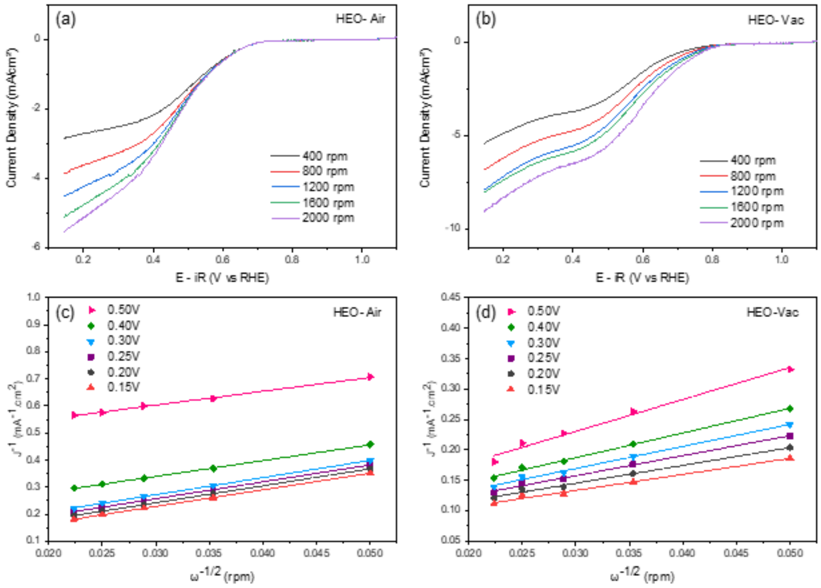

Figure S2. Linear Sweep Voltammetry of (a) HEO- Air, and (b) HEO- Vac at different rotation speeds (400, 800, 1200, 1600, 2000 rpm), Koutecky- Levich (K-L) plots of (c) HEO-Air, and (d) HEO- Vac at 0.50, 0.40, 0.30, 0.25, 0.20, and 0.15 V.

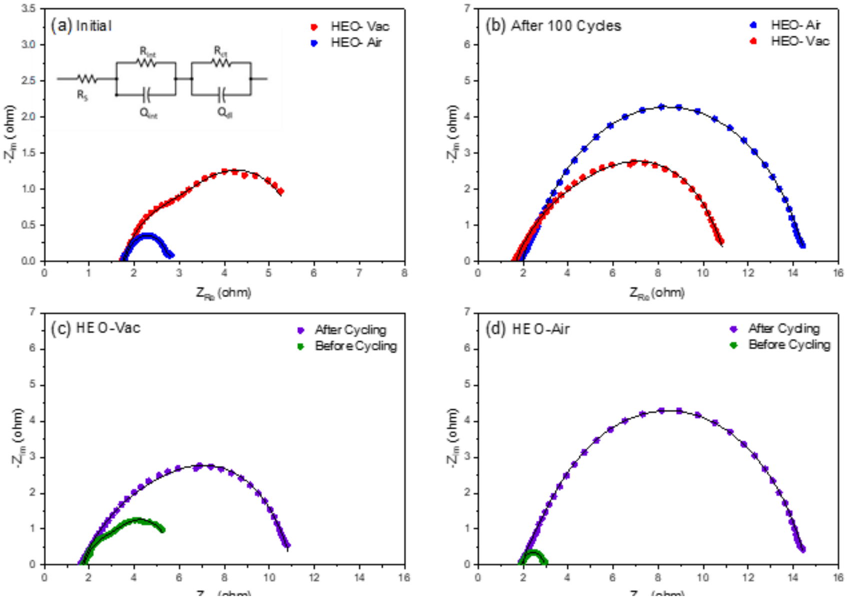

Figure S3. Electrochemical Impedance Spectroscopy (EIS) of (a) HEO-Vac and HEO-Air based Zn-Air batteries before cyclic charge-discharge (Beginning of life) (b) HEO-Vac and HEO-Air

based Zn-Air batteries after 100 cycles of cyclic charge-discharge (End of life) (c) HEO-Vac before and after cycling (d) HEO-Air before and after cycling.

Table S5. The parameters representing the electrical components in the equivalent circuit, determined through EIS for HEO-Vac and HEO-Air based Zn-Air batteries before (BOL EIS) and after (EOL EIS) cycling charge discharge.

	HEO- Vac (BOL)	HEO- Air (EOL)	HEO- Vac (EOL)	HEO- Air (EOL)
R_s (Ω)	1.746	1.560	1.661	2.906
R_{int} (Ω)	1.802	0.457	3.771	5.129
R_{ct} (Ω)	2.200	0.813	5.577	9.620
Q_{int} ($S.s^n$)	$7.389*10^{-4}$	2.076	$6.770*10^{-3}$	$8.520*10^{-3}$
Q_{dl} ($S.s^n$)	$3.01*10^{-1}$	$4.449*10^{-4}$	$4.920*10^{-3}$	$1.694*10^{-3}$

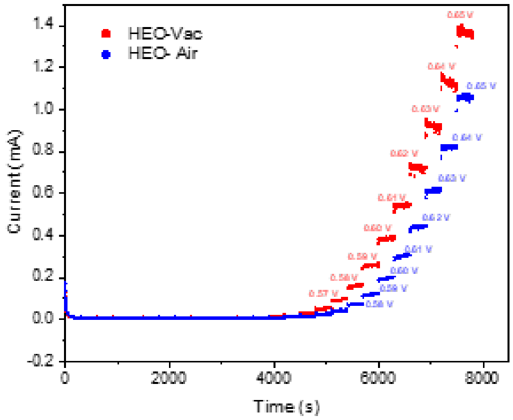

Figure S4. Chronoamperometry to obtain Tafel slopes for HEO-Air and HEO-Vac

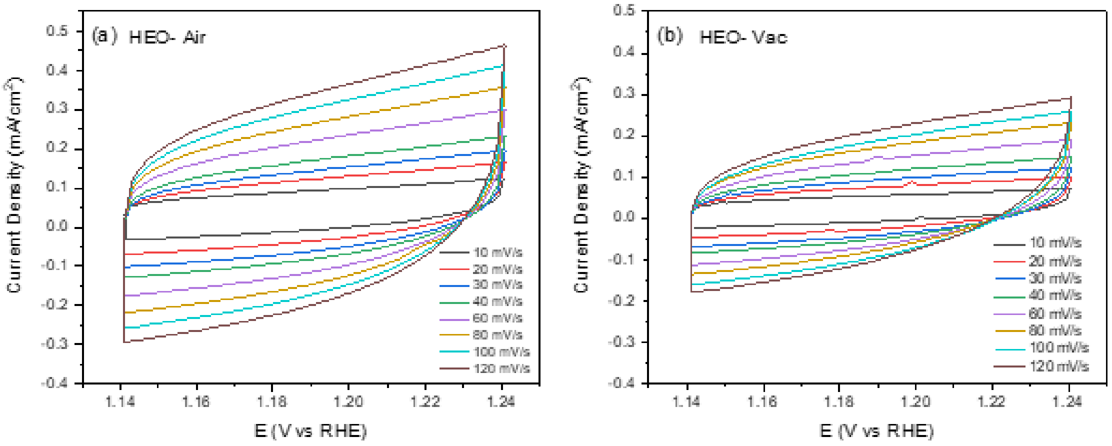

Figure S5. Cyclic Voltammetry scans of (a) HEO- Air, and (b) HEO- Vac at 10, 20, 30, 40, 60, 80, 100, and 120 mV s^{-1} scan rates.

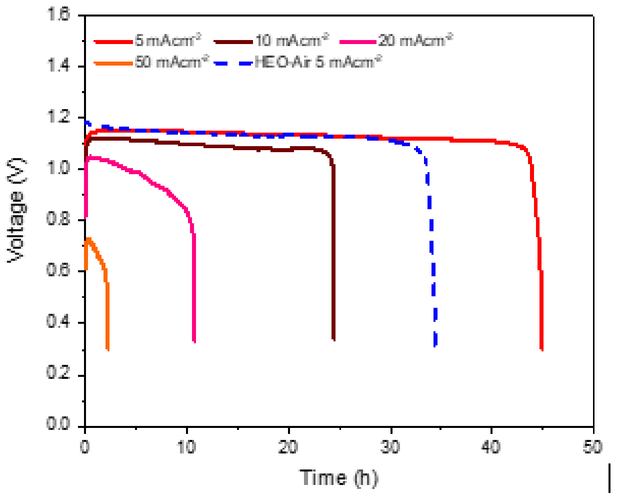

Figure S6. Voltage (V) vs time (h) for HEO-Vac based Zn-Air battery at 5, 10, 20, and 50 mA.cm^{-2} current densities, and
